# 2-(2-Fluoro­phen­yl)-*N*-(1,3-thia­zol-2-yl)acetamide

**DOI:** 10.1107/S1600536812032989

**Published:** 2012-07-28

**Authors:** Hoong-Kun Fun, Ching Kheng Quah, Prakash S. Nayak, B. Narayana, B. K. Sarojini

**Affiliations:** a X-ray Crystallography Unit, School of Physics, Universiti Sains Malaysia, 11800 USM, Penang, Malaysia; b Department of Studies in Chemistry, Mangalore University, Mangalagangotri 574 199, India; c Department of Chemistry, P. A. College of Engineering, Nadupadavu, Mangalore 574 153, India

## Abstract

In the title compound, C_11_H_9_FN_2_OS, the 1,3-thia­zole ring is planar (r.m.s. deviation = 0.007 Å) and forms a dihedral angle of 73.75 (5)° with the benzene ring. In the crystal, mol­ecules are linked *via* pairs of N—H⋯N and C—H⋯F hydrogen bonds into chains along [100].

## Related literature

For general background to the title compound and for related structures, see: Fun *et al.* (2011*a* ▶,*b* ▶, 2012*a* ▶,*b* ▶). For the stability of the temperature controller used in the data collection, see: Cosier & Glazer (1986 ▶). For standard bond lengths, see: Allen *et al.* (1987 ▶).
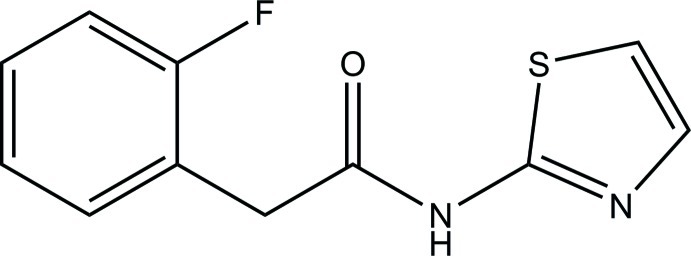


## Experimental

### 

#### Crystal data

C_11_H_9_FN_2_OS
*M* *_r_* = 236.26
Monoclinic, 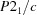

*a* = 11.9043 (13) Å
*b* = 5.2969 (6) Å
*c* = 16.4579 (18) Å
β = 90.397 (3)°
*V* = 1037.7 (2) Å^3^
*Z* = 4
Mo *K*α radiation
μ = 0.30 mm^−1^
*T* = 100 K
0.26 × 0.18 × 0.12 mm

#### Data collection

Bruker SMART APEXII DUO CCD area-detector diffractometer
Absorption correction: multi-scan (*SADABS*; Bruker, 2009 ▶) *T*_min_ = 0.924, *T*_max_ = 0.965
12231 measured reflections
3722 independent reflections
3092 reflections with *I* > 2σ(*I*)
*R*_int_ = 0.030

#### Refinement

*R*[*F* ^2^ > 2σ(*F* ^2^)] = 0.034
*wR*(*F* ^2^) = 0.090
*S* = 1.05
3722 reflections
149 parameters
H atoms treated by a mixture of independent and constrained refinement
Δρ_max_ = 0.47 e Å^−3^
Δρ_min_ = −0.26 e Å^−3^

### 

Data collection: *APEX2* (Bruker, 2009 ▶); cell refinement: *SAINT* (Bruker, 2009 ▶); data reduction: *SAINT*; program(s) used to solve structure: *SHELXTL* (Sheldrick, 2008 ▶); program(s) used to refine structure: *SHELXTL*; molecular graphics: *SHELXTL*; software used to prepare material for publication: *SHELXTL* and *PLATON* (Spek, 2009 ▶).

## Supplementary Material

Crystal structure: contains datablock(s) global, I. DOI: 10.1107/S1600536812032989/rz2790sup1.cif

Structure factors: contains datablock(s) I. DOI: 10.1107/S1600536812032989/rz2790Isup2.hkl

Supplementary material file. DOI: 10.1107/S1600536812032989/rz2790Isup3.cml

Additional supplementary materials:  crystallographic information; 3D view; checkCIF report

## Figures and Tables

**Table 1:** Hydrogen-bond geometry (Å, °)

*D*—H⋯*A*	*D*—H	H⋯*A*	*D*⋯*A*	*D*—H⋯*A*
N2—H1*N*2⋯N1^i^	0.845 (16)	2.095 (16)	2.9376 (14)	175.0 (16)
C10—H10*A*⋯F1^ii^	0.95	2.51	3.4071 (14)	157

Symmetry codes: (i) 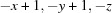; (ii) 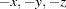.

**Fig. 1.**
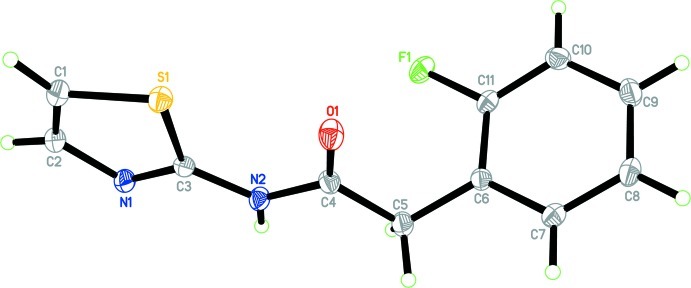
The molecular structure of the title compound showing 50% probability displacement ellipsoids for non-H atoms.

**Fig. 2.**
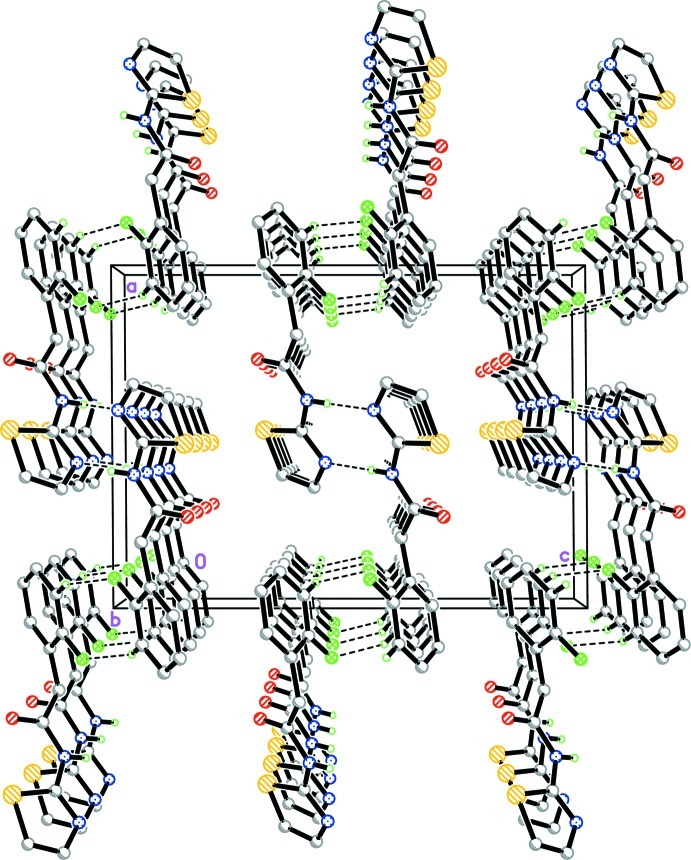
The crystal structure of the title compound, viewed along the b axis. H atoms not involved in hydrogen bonds (dashed lines) have been omitted for clarity.

## supplementary crystallographic information

### Comment

In continuation of our work on synthesis of amides (Fun *et al.*, 2011*a*, 2011*b*, 2012*a*, 2012*b*), we report herein the crystal structure of the title compound.

In the title molecule (Fig. 1), the thiazol-2-yl ring (S1/N1/C1-C3) is nearly planar (r.m.s. deviation = 0.007 Å) and forms a dihedral angle of 73.75 (5)° with the benzene ring (C6-C11). Bond lengths (Allen *et al.*, 1987) and angles are within normal ranges and are comparable to those reported for related structures (Fun *et al.*, 2011*a*, 2011*b*, 2012*a*, 2012*b*). In the crystal structure (Fig. 2), molecules are linked *via* pairs of intermolecular N2–H1N2···N1 and C10–H10A···F1 hydrogen bonds (Table 1) into one-dimensional chains along [100].

### Experimental

2-Fluorolphenylacetic acid (0.154 g, 1 mmol), 2-aminothiazole (0.1 g, 1 mmol) and 1-ethyl-3-(3-dimethylaminopropyl)-carbodiimide hydrochloride (1.0 g, 0.01 mol) were dissolved in dichloromethane (20 mL). The mixture was stirred in presence of triethylamine at 273 K for about 3 h. The contents were poured into 100 mL of ice-cold aqueous hydrochloric acid with stirring and was then extracted thrice with dichloromethane. The organic layer was washed with saturated NaHCO_3_ solution and brine solution, dried and concentrated under reduced pressure to give the title compound. Single crystals were grown from acetone and toluene (1:1 *v*/*v*) mixture by the slow evaporation method (m.p.: 453-455 K).

### Refinement

Atom H1N2 was located in a difference Fourier map and refined freely [N–H = 0.846 (17) Å]. The remaining H atoms were positioned geometrically and refined using a riding model with C–H = 0.95 or 0.99 Å and *U*_iso_(H) = 1.2 *U*_eq_(C).

### Crystal data

**Table:**

C_11_H_9_FN_2_OS	*F*(000) = 488
*M**_r_* = 236.26	*D*_x_ = 1.512 Mg m^−^^3^
Monoclinic, *P*2_1_/*c*	Mo *K*α radiation, λ = 0.71073 Å
Hall symbol: -P 2ybc	Cell parameters from 4325 reflections
*a* = 11.9043 (13) Å	θ = 4.0–32.5°
*b* = 5.2969 (6) Å	µ = 0.30 mm^−^^1^
*c* = 16.4579 (18) Å	*T* = 100 K
β = 90.397 (3)°	Block, colourless
*V* = 1037.7 (2) Å^3^	0.26 × 0.18 × 0.12 mm
*Z* = 4	

### Data collection

**Table:**

Bruker SMART APEXII DUO CCD area-detector diffractometer	3722 independent reflections
Radiation source: fine-focus sealed tube	3092 reflections with *I* > 2σ(*I*)
Graphite monochromator	*R*_int_ = 0.030
φ and ω scans	θ_max_ = 32.6°, θ_min_ = 3.0°
Absorption correction: multi-scan (*SADABS*; Bruker, 2009)	*h* = −17→18
*T*_min_ = 0.924, *T*_max_ = 0.965	*k* = −8→7
12231 measured reflections	*l* = −24→24

### Refinement

**Table:**

Refinement on *F*^2^	Primary atom site location: structure-invariant direct methods
Least-squares matrix: full	Secondary atom site location: difference Fourier map
*R*[*F*^2^ > 2σ(*F*^2^)] = 0.034	Hydrogen site location: inferred from neighbouring sites
*wR*(*F*^2^) = 0.090	H atoms treated by a mixture of independent and constrained refinement
*S* = 1.05	*w* = 1/[σ^2^(*F*_o_^2^) + (0.0396*P*)^2^ + 0.3919*P*] where *P* = (*F*_o_^2^ + 2*F*_c_^2^)/3
3722 reflections	(Δ/σ)_max_ = 0.001
149 parameters	Δρ_max_ = 0.47 e Å^−^^3^
0 restraints	Δρ_min_ = −0.26 e Å^−^^3^

### Special details

**Table:**

Experimental. The crystal was placed in the cold stream of an Oxford Cryosystems Cobra open-flow nitrogen cryostat (Cosier & Glazer, 1986) operating at 100.0 (1) K.
Geometry. All esds (except the esd in the dihedral angle between two l.s. planes) are estimated using the full covariance matrix. The cell esds are taken into account individually in the estimation of esds in distances, angles and torsion angles; correlations between esds in cell parameters are only used when they are defined by crystal symmetry. An approximate (isotropic) treatment of cell esds is used for estimating esds involving l.s. planes.
Refinement. Refinement of F^2^ against ALL reflections. The weighted R-factor wR and goodness of fit S are based on F^2^, conventional R-factors R are based on F, with F set to zero for negative F^2^. The threshold expression of F^2^ > 2sigma(F^2^) is used only for calculating R-factors(gt) etc. and is not relevant to the choice of reflections for refinement. R-factors based on F^2^ are statistically about twice as large as those based on F, and R- factors based on ALL data will be even larger.

### Fractional atomic coordinates and isotropic or equivalent isotropic displacement parameters (Å^2^)

**Table:**

	*x*	*y*	*z*	*U*_iso_*/*U*_eq_	
F1	0.11704 (6)	0.19317 (14)	0.03863 (4)	0.02223 (16)	
S1	0.48132 (2)	0.00859 (5)	0.172709 (16)	0.01629 (7)	
O1	0.28432 (7)	0.26500 (17)	0.18303 (5)	0.01993 (17)	
N1	0.57205 (8)	0.22624 (18)	0.04881 (6)	0.01618 (17)	
N2	0.39938 (7)	0.40844 (19)	0.08374 (6)	0.01519 (17)	
C1	0.60857 (9)	−0.1033 (2)	0.13824 (7)	0.0184 (2)	
H1A	0.6491	−0.2397	0.1618	0.022*	
C2	0.64234 (9)	0.0308 (2)	0.07290 (7)	0.0176 (2)	
H2A	0.7101	−0.0065	0.0452	0.021*	
C3	0.48369 (8)	0.2335 (2)	0.09615 (6)	0.01391 (18)	
C4	0.30210 (9)	0.4142 (2)	0.12773 (6)	0.01507 (19)	
C5	0.22188 (9)	0.6224 (2)	0.10236 (7)	0.0186 (2)	
H5A	0.2419	0.7787	0.1321	0.022*	
H5B	0.2314	0.6559	0.0436	0.022*	
C6	0.10050 (8)	0.5610 (2)	0.11813 (6)	0.01376 (18)	
C7	0.03141 (9)	0.7177 (2)	0.16408 (6)	0.01611 (19)	
H7A	0.0621	0.8652	0.1884	0.019*	
C8	−0.08201 (9)	0.6617 (2)	0.17497 (7)	0.0185 (2)	
H8A	−0.1279	0.7711	0.2062	0.022*	
C9	−0.12777 (9)	0.4461 (2)	0.14008 (7)	0.0199 (2)	
H9A	−0.2049	0.4076	0.1478	0.024*	
C10	−0.06080 (9)	0.2858 (2)	0.09371 (7)	0.0181 (2)	
H10A	−0.0912	0.1380	0.0693	0.022*	
C11	0.05083 (9)	0.3483 (2)	0.08436 (6)	0.01515 (19)	
H1N2	0.4086 (14)	0.506 (3)	0.0437 (10)	0.027 (4)*	

### Atomic displacement parameters (Å^2^)

**Table:**

	*U*^11^	*U*^22^	*U*^33^	*U*^12^	*U*^13^	*U*^23^
F1	0.0244 (3)	0.0189 (3)	0.0235 (3)	0.0046 (3)	0.0039 (3)	−0.0076 (3)
S1	0.01630 (12)	0.01661 (13)	0.01597 (12)	0.00145 (10)	0.00162 (8)	0.00255 (9)
O1	0.0171 (3)	0.0237 (4)	0.0191 (3)	0.0032 (3)	0.0045 (3)	0.0065 (3)
N1	0.0135 (4)	0.0153 (4)	0.0198 (4)	0.0008 (3)	0.0028 (3)	0.0007 (3)
N2	0.0135 (4)	0.0149 (4)	0.0172 (4)	0.0017 (3)	0.0033 (3)	0.0031 (3)
C1	0.0156 (4)	0.0175 (5)	0.0220 (5)	0.0029 (4)	−0.0010 (4)	0.0016 (4)
C2	0.0135 (4)	0.0173 (5)	0.0222 (5)	0.0010 (4)	0.0010 (4)	−0.0003 (4)
C3	0.0134 (4)	0.0128 (4)	0.0155 (4)	−0.0010 (4)	0.0007 (3)	−0.0006 (3)
C4	0.0129 (4)	0.0163 (5)	0.0160 (4)	−0.0006 (4)	0.0015 (3)	−0.0002 (4)
C5	0.0141 (4)	0.0164 (5)	0.0254 (5)	0.0010 (4)	0.0032 (4)	0.0036 (4)
C6	0.0130 (4)	0.0138 (4)	0.0145 (4)	0.0007 (4)	0.0010 (3)	0.0011 (3)
C7	0.0176 (4)	0.0145 (5)	0.0163 (4)	0.0015 (4)	0.0010 (3)	−0.0016 (4)
C8	0.0177 (5)	0.0195 (5)	0.0186 (5)	0.0054 (4)	0.0042 (4)	0.0010 (4)
C9	0.0136 (4)	0.0216 (5)	0.0244 (5)	0.0008 (4)	0.0015 (4)	0.0046 (4)
C10	0.0168 (4)	0.0156 (5)	0.0219 (5)	−0.0012 (4)	−0.0032 (4)	0.0008 (4)
C11	0.0169 (4)	0.0139 (5)	0.0147 (4)	0.0030 (4)	0.0015 (3)	−0.0017 (4)

### Geometric parameters (Å, º)

**Table:**

F1—C11	1.3679 (12)	C5—C6	1.5054 (15)
S1—C1	1.7262 (11)	C5—H5A	0.9900
S1—C3	1.7344 (11)	C5—H5B	0.9900
O1—C4	1.2248 (13)	C6—C11	1.3866 (15)
N1—C3	1.3139 (13)	C6—C7	1.3951 (14)
N1—C2	1.3872 (15)	C7—C8	1.3952 (15)
N2—C4	1.3704 (13)	C7—H7A	0.9500
N2—C3	1.3802 (14)	C8—C9	1.3878 (17)
N2—H1N2	0.846 (17)	C8—H8A	0.9500
C1—C2	1.3524 (16)	C9—C10	1.3956 (16)
C1—H1A	0.9500	C9—H9A	0.9500
C2—H2A	0.9500	C10—C11	1.3791 (15)
C4—C5	1.5156 (16)	C10—H10A	0.9500
			
C1—S1—C3	88.76 (5)	C4—C5—H5B	108.9
C3—N1—C2	109.65 (9)	H5A—C5—H5B	107.7
C4—N2—C3	123.58 (9)	C11—C6—C7	116.69 (10)
C4—N2—H1N2	120.8 (12)	C11—C6—C5	120.92 (9)
C3—N2—H1N2	115.4 (12)	C7—C6—C5	122.34 (10)
C2—C1—S1	110.32 (9)	C6—C7—C8	121.19 (10)
C2—C1—H1A	124.8	C6—C7—H7A	119.4
S1—C1—H1A	124.8	C8—C7—H7A	119.4
C1—C2—N1	115.93 (10)	C9—C8—C7	119.94 (10)
C1—C2—H2A	122.0	C9—C8—H8A	120.0
N1—C2—H2A	122.0	C7—C8—H8A	120.0
N1—C3—N2	121.08 (10)	C8—C9—C10	120.20 (10)
N1—C3—S1	115.33 (8)	C8—C9—H9A	119.9
N2—C3—S1	123.59 (8)	C10—C9—H9A	119.9
O1—C4—N2	121.95 (10)	C11—C10—C9	117.99 (11)
O1—C4—C5	124.23 (9)	C11—C10—H10A	121.0
N2—C4—C5	113.82 (9)	C9—C10—H10A	121.0
C6—C5—C4	113.50 (9)	F1—C11—C10	118.45 (10)
C6—C5—H5A	108.9	F1—C11—C6	117.56 (9)
C4—C5—H5A	108.9	C10—C11—C6	123.98 (10)
C6—C5—H5B	108.9		
			
C3—S1—C1—C2	−0.63 (9)	C4—C5—C6—C11	−57.78 (14)
S1—C1—C2—N1	1.22 (13)	C4—C5—C6—C7	124.88 (11)
C3—N1—C2—C1	−1.27 (14)	C11—C6—C7—C8	−0.15 (15)
C2—N1—C3—N2	−178.59 (10)	C5—C6—C7—C8	177.30 (10)
C2—N1—C3—S1	0.73 (12)	C6—C7—C8—C9	0.31 (17)
C4—N2—C3—N1	175.57 (10)	C7—C8—C9—C10	−0.36 (17)
C4—N2—C3—S1	−3.70 (15)	C8—C9—C10—C11	0.25 (17)
C1—S1—C3—N1	−0.07 (9)	C9—C10—C11—F1	−179.62 (10)
C1—S1—C3—N2	179.23 (10)	C9—C10—C11—C6	−0.09 (17)
C3—N2—C4—O1	1.54 (17)	C7—C6—C11—F1	179.57 (9)
C3—N2—C4—C5	−179.49 (10)	C5—C6—C11—F1	2.08 (15)
O1—C4—C5—C6	−29.04 (16)	C7—C6—C11—C10	0.04 (16)
N2—C4—C5—C6	152.01 (10)	C5—C6—C11—C10	−177.45 (10)

### Hydrogen-bond geometry (Å, º)

**Table:**

*D*—H···*A*	*D*—H	H···*A*	*D*···*A*	*D*—H···*A*
N2—H1*N*2···N1^i^	0.845 (16)	2.095 (16)	2.9376 (14)	175.0 (16)
C10—H10*A*···F1^ii^	0.95	2.51	3.4071 (14)	157

Symmetry codes: (i) −*x*+1, −*y*+1, −*z*; (ii) −*x*, −*y*, −*z*.
